# May Homiletic Review

**Published:** 1889-06

**Authors:** 


THE MAY HOMILETIC REVIEW has an admirable paper on Hugh Latima, the Homilist, by Prof. Hunt of Princeton. Dr. A. T. Pierson discusses Church Talent in a vigorous and somewhat original fashion. Prof. Welsh of Auburn Seminary shows the relation of doctrine and duty in a very clear and forcible manner. The nervous system and sin is the theme of Dr. Stone's second paper on Body and Mind in Christian Life. Dr. John Hall in a characteristic paper answers the question, What is the Ministry? What its Work? The preachers of the Old Testament is another suggestive article. The sermonic section is unusually rich and varied, and Living Issues are unusually full and vigor ous, and on the whole the number is one of full average ability and interest.

Published by Funk & Wagnalls, 18 and 20 Astor Place, New York. $3.00 per year; 30 cents per single number.

